# Changes in oxygen supply–demand balance during induction of general anesthesia: an exploratory study using remimazolam

**DOI:** 10.1007/s00540-024-03362-0

**Published:** 2024-06-06

**Authors:** Kenya Yarimizu, Yu Onodera, Hiroto Suzuki, Masaki Nakane, Kaneyuki Kawamae

**Affiliations:** 1 Department of Anesthesiology, Yamagata University Hospital, 2-2-2 Iida-Nishi, Yamagata, Yamagata 990-9585 Japan; 2 Yamagata City Miyuki Hospital, Yamagata, Japan; 3 Department of Emergency and Critical Care Medicine, Yamagata University Hospital, Yamagata, Japan; 4 Ohta-Nishinouti Hospital, Fukushima, Japan

**Keywords:** Remimazolam, Oxygen consumption, Oxygen supply, Oxygen supply–demand balance, General anesthesia

## Abstract

**Purpose:**

This study was performed to evaluate the changes in oxygen supply–demand balance during induction of general anesthesia using an indirect calorimeter capable of measuring oxygen consumption (VO_2_) and carbon dioxide production (VCO_2_).

**Methods:**

This study included patients scheduled for surgery in whom remimazolam was administered as a general anesthetic. VO_2_ and VCO_2_ were measured at different intervals: upon awakening (T1), 15 min after tracheal intubation (T2), and 1 h after T2 (T3). Oxygen delivery (DO_2_) was calculated simultaneously with these measurements. VO_2_ was ascertained using an indirect calorimeter and further calculated using vital signs, among other factors. DO_2_ was derived from cardiac output and arterial blood gas analysis performed with an arterial pressure-based cardiac output measurement system.

**Results:**

VO_2_, VCO_2_, and DO_2_ decreased significantly from T1 to T2 and T3 [VO_2_/body surface area (BSA) (ml/min/m^2^): T1, 130 (122–146); T2, 107 (83–139); T3, 97 (93–121); *p* = 0.011], [VCO_2_/BSA (ml/min/m^2^): T1, 115 (105–129); T2, 90 (71–107); T3, 81 (69–101); *p* = 0.011], [DO_2_/BSA (ml/min/m^2^): T1, 467 (395–582); T2, 347 (286–392); T3, 382 (238–414); *p* = 0.0020]. Among the study subjects, a subset exhibited minimal reduction in VCO_2_. Although the respiratory frequency was titrated on the basis of end-tidal CO_2_ levels, there was no significant difference between the groups.

**Conclusion:**

General anesthetic induction with remimazolam decreased VO_2_, VCO_2_, and DO_2_.

## Introduction

A key objective in anesthesia and intensive care is preservation of oxygen homeostasis [1]. Administration of general anesthesia transfers homeostasis regulation from the patient’s innate physiological regulatory mechanisms to the anesthesiologist. Therefore, careful control of anesthetic procedures, particularly systemic management of general anesthetics, is necessary. When oxygen delivery (DO_2_) to tissues decreases, in vivo mechanisms that regulate the oxygen-carrying capacity [2] and shut down unneeded cellular operations are activated [3]. However, when tissue oxygen levels fall below a particular threshold, malfunction of intracellular metabolic processes may result in life-threatening shock [4, 5].

DO_2_ to tissues is proportional to the product of arterial blood oxygen content and cardiac output [6]. Critical DO_2_ levels [DO_2_(crit)] are those that cause cellular and organ malfunction and irreversible alterations that can lead to cell death [7, 8]. There is no definitive standard for DO_2_(crit). Oxygen consumption (VO_2_) in vivo is independent of DO_2_ over a broad range of values because the oxygen extraction rate (ERO_2_), representing the ratio of VO_2_ to DO_2_, can accommodate changes in DO_2_ [9]. However, when oxygen supply falls below DO_2_(crit), cellular oxygen utilization becomes dependent on the oxygen supply [10, 11].

The balance of oxygen supply is not solely dependent on the amount of oxygen delivered. The effect of carbon dioxide produced by tissue metabolism (VCO_2_) also plays a role. Further increases in VCO_2_ can lead to greater increases in the partial pressure of carbon dioxide, potentially resulting in acidosis. The carbon dioxide dissociation curve changes with alterations in base excess, hemoglobin concentration, and oxygen saturation (Haldane effect), as outlined in a previous report [12]. These factors can alter the venoarterial difference in carbon dioxide tension, even without changes in the R/Q (VCO_2_/VO_2_) or tissue oxygenation [13].

During induction of general anesthesia, rapid changes occur in the patient’s hemodynamic and respiratory condition [14]. Anesthetic-induced circulatory depression decreases cardiac output and DO_2_, whereas sedative medications [15], muscle relaxants [16], and changes in body temperature influence VO_2_ [17]. Consequently, both DO_2_ and VO_2_ drop during general anesthesia, but their effects and associations are unknown [18].

Selection of medications is a crucial aspect of anesthetic management. Remimazolam is an ultrashort-acting sedative/anesthetic benzodiazepine agonist with high affinity for the benzodiazepine-binding site on the gamma-aminobutyric acid receptor. This drug is characterized by the safety of benzodiazepines (hemodynamic stability) and excellent controllability (quick induction of anesthesia and recovery of cognitive functions) [19–21].

Hypotensive episodes requiring vasoconstrictor treatment during general anesthesia can influence the oxygen supply–demand balance [22–24]. DO_2_ and VO_2_ during general anesthesia may have been exaggerated in prior studies that reported the balance between oxygen supply and demand, as the use of drugs that increase blood pressure may have increased oxygen supply and oxygen consumption, which could have been overestimated. Therefore, we expected that using remimazolam, which is more hemodynamically stable than typical general anesthetics [25], would facilitate analysis of alterations in the oxygen supply–demand balance generated by general anesthesia without vasoconstrictor treatment.

We conducted an exploratory investigation to determine the effects of remimazolam on the balance between oxygen supply and demand during general anesthetic induction by monitoring VO_2_, VCO_2_, and DO_2_. Our objective was twofold: first, to determine the impact of remimazolam on the balance between DO_2_ and VO_2_ during general anesthetic induction; and second, to explore the in vivo changes associated with changes in the oxygen supply–demand balance during arousal and after the induction of general anesthesia.

## Methods

This research was approved by Yamagata University School of Medicine Ethics Review Board (Approval No. 2020–375; Approval Date: April 7, 2021). All participants provided written informed consent. Before study commencement, registration in the University Hospital Medical Information Network was completed (Study ID: UMIN000043879; Reception Number: R000050079; Publication Date: April 10, 2021).

The participants were surgical patients who underwent general anesthesia with intubation in our institution. The eligibility requirement was an age of 18–85 years. The exclusion criteria were allergy to any component of remimazolam; history of central nervous system disease (e.g., cerebral infarction or hemorrhage), neuromuscular disease (e.g., myasthenia gravis), uncontrolled heart or respiratory failure, renal failure (serum creatinine ≥ 2 mg/dl), or liver failure (aspartate transaminase/alanine transaminase ratio 2.5 × the upper limit of normal); and inability to provide informed consent. Patients requiring postural adjustment or isolated lung ventilation, those undergoing head-and-neck surgery and emergency procedures, and those with hemorrhage that might influence cardiac performance were also excluded. Patients receiving epidural anesthesia were not excluded.

### Anesthetic management and monitoring

A transcutaneous oxygen saturation monitor, electrocardiogram device, noninvasive arterial pressure monitor, bispectral index (BIS) monitor (BIS XP; Aspect Medical Systems, Newton, MA, USA), and peripheral venous and arterial lines were applied upon entering the operating room. Before general anesthetic induction, the supraorbital forehead temperature was recorded using a zero-heat-flux (ZHF) thermometer (SpotOn; 3 M, Saint Paul, MN, USA). After general anesthetic induction, a urethral catheter with a bladder temperature sensor (BARD Silver TSC Tray; Becton, Dickinson and Company, Franklin Lakes, NJ, USA) was placed.

Before anesthetic induction, VO_2_ and VCO_2_ were measured with an indirect calorimeter (CCM Express; MGC Diagnostics, Saint Paul, MN, USA), and oxygen supply was simultaneously estimated (T1). Following preoxygenation, remifentanil (0.2–0.5 μg/kg/min) and remimazolam (Anerem 50 mg; Mundipharma K.K., Tokyo, Japan) (12 mg/kg/h) were administered. Upon confirming loss of consciousness, the remimazolam dose was lowered to 1.0 mg/kg/h and subsequently adjusted to maintain a BIS of 40–60. Rocuronium was administered at 0.8 mg/kg, followed by intubation 2–3 min later. Post-intubation, the remifentanil dosage was decreased to 0.1 μg/kg/min and maintained. In addition, the dose of remifentanil was increased to 0.2–0.4 μg/kg/min after T2 and maintained until surgery. The body surface heating system was set to 38 °C in areas not covered by the surgical field. The heating device was not applied above the patient’s neck. The crystalloid solution administration speed was not changed from T1–T3.

Systolic arterial pressure of < 80 mmHg was defined as hypotension. For hypotension, 0.1 mg of phenylephrine and 4 mg of ephedrine were administered when the pulse rate was ≥ 80 and ≤ 79 beats/min, respectively. These participants were excluded from the study.

Inspiratory oxygenation was set to 45%, the tidal volume for ventilation was 8 ml/kg [male body weight (kg): 50 + 0.91(height (cm) − 152.4); female body weight (kg): 45.5 + 0.91(height (cm) − 152.4)] [26], the end-expiratory positive-pressure ventilation was 5 cmH_2_O, and the respiratory rate was adjusted to an end-tidal carbon dioxide concentration of 35–40 mmHg. A Perseus A500 ventilator (Dräger, Lübeck, Germany) was used.

Using an indirect calorimeter, the metabolic rate was assessed 15 min after tracheal intubation and stabilization of the hemodynamic and respiratory state (T2), and again 1 h after T2 (T3).

The oxygen supply–demand balance was determined using the indirect calorimeter measurements of VO_2_ and VCO_2_. The indirect calorimeter measured VO_2_ and VCO_2_ based on the oxygen or carbon dioxide concentration and the breathing rate for inspiratory and expiratory air [27].

Cardiac output was measured with a FloTrac (Edwards Lifesciences, Irvine, CA, USA) and combined with the arterial blood gas measurements to calculate DO_2_ as follows [7]:

DO_2_ = [1.34 × Hb × SaO_2_ + (0.003 × PaO_2_)] × cardiac output × 10,

where Hb is hemoglobin (g/dl), SaO_2_ is arterial oxyhemoglobin saturation (%), and PaO_2_ is arterial oxygen tension (mmHg). The cardiac output measured by the FloTrac was calculated as the product of stroke volume and heart rate.

When the respiratory and circulatory dynamics were steady, measurements were collected once, and the 3 min mean of the measurements was used. The variables are displayed as median (interquartile range).

### Outcomes

The primary endpoint was the changes in VO_2_ and VCO_2_ evaluated by indirect calorimetry before and after general anesthetic induction, as well as DO_2_ derived from cardiac output.

The secondary endpoints included various vital signs, such as blood pressure, heart rate, cardiac output, and BIS. We also studied indices of the oxygen supply–demand balance before and after general anesthetic induction, namely ERO_2_, R/Q, double product (heart rate × systemic blood pressure) [28], and VO_2_ using the LaFarge–Miettinen prediction equation (LMVO_2_) for men and women [men: LMVO_2_/body surface area (BSA) = 138.1 − (11.19 × log age) + (0.378 × heart rate), women: LMVO_2_/BSA = 138.1 − (17.04 × log age) + (0.378 × heart rate)] [29].

Patients were divided into two cohorts on the basis of VCO_2_ values at T2, which is a metabolic byproduct and indicator of respiratory function [30]. VCO_2_ per body surface area of 96.3 ml/min/m^2^ was defined as the boundary value, with patients exceeding this value comprising the high VCO_2_ group and those below this value comprising the low VCO_2_ group. This boundary value was determined from the resting VO_2_ value [31], the predicted decrease in VO_2_ under general anesthesia [23], and a respiratory quotient of 1. The cohort division aimed to elucidate differences in oxygen metabolic parameters, such as VO_2_ and VCO_2_, between wakefulness and after induction of anesthesia.

### Sample size and statistical analyses.

The sample size was determined based on the standardized mean difference of 1.31 and standardized difference of 25 as previously described [32]. A sample size of 11 was needed to detect a > 30% change in VO_2_ with a significance level of 0.05 (two-tailed) and a power of 0.80, as previously reported [23]. Because indirect calorimetry measurement involves skill, the target number of patients was set at 22, assuming that measurement errors would occur in approximately 50% of cases.

The Friedman test for comparing measurement data at three sites and the Wilcoxon rank-sum test for comparing two groups were used for statistical analyses. Two-tailed tests were conducted, and *p* < 0.05 was considered significant.

All statistical analyses were conducted using GraphPad Prism version 5.00 (GraphPad Software, San Diego, CA, USA).

On the consent form, the participants were informed that they would receive remimazolam for general anesthesia. The anesthesiologist in charge of each case also performed the measurements and analyzed the results. One anesthesiologist performed the measurements with the assistance of one anesthesia assistant.

The figures were created using GraphPad Prism version 5.00 (GraphPad Software Inc., San Diego, CA, USA) and Microsoft PowerPoint (Microsoft Corporation, Redmond, WA, USA).

## Results

**Fig. 1:**
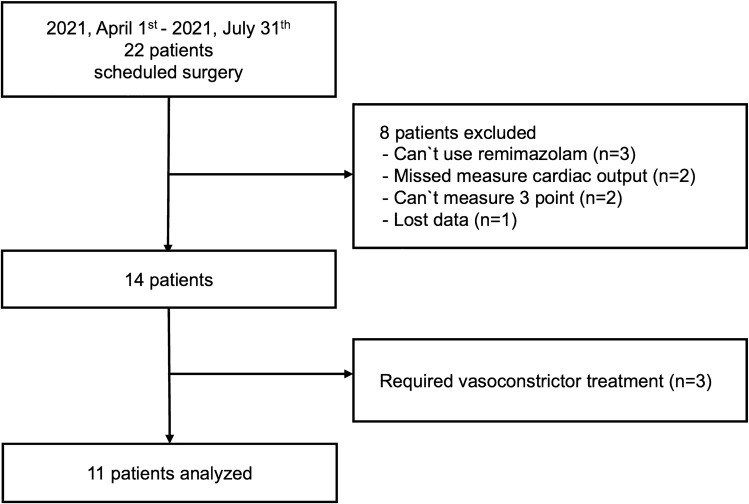
Diagram illustrating the included patients. The study involved 11 patients after omitting those who met the exclusion criteria and those who received vasoconstrictor treatment

Twenty-two patients were enrolled from April 2021 to August 2021; of these, 11 patients were included (Fig. 1). The patients’ demographics were characterized by a median age of 67 years and no sex bias (Table 1). Heart rate, blood pressure, and hemoglobin decreased markedly from T1–T2 and T3 (Table 2).

**Table 1:** Patient characteristics

Variable	*n* = 11
Age (year)	67 (56–79)
Male (*n*, %)	6 (54.5)
Height (m)	1.61 (1.58–1.67)
Body weight (kg)	59.6 (53.9–69.1)
Body mass index (kg/m^2^)	21.9 (20.1–27.8)
Body surface area (m^2^)	1.62 (1.54–1.79)
ASA-PS (1/2/3)	2 /8 /1
Comorbidities	
Hypertension (*n*, %)	8 (72.7)
Diabetes mellitus (*n*, %)	2 (18.2)
Medication	
Beta-blocker (*n*, %)	1 (9.0)
Charlson Comorbidity Index score	2 (1.5–2)
Age-adjusted Charlson comorbidity index score	6 (5.5–6.0)
Preoperative hemoglobin (g/dl)	12.6 (11.6–13.3)
Preoperative creatinine (mg/dl)	0.76 (0.61–0.86)
Operation for cancer (*n*, %)	8 (72.7)
Type of surgery	
Abdominal surgery (*n*, %)	8 (72.7)
Cardiovascular surgery (*n*, %)	2 (18.1)
Mastectomy (*n*, %)	1 (9.0)

Data are represented as the median (interquartile range) or number of patients

*ASA-PS* American society of anesthesiologists physical status

**Table 2:** Value as measured at each phase of the measurement

Variable	T1	T2	T3	p
Temperature by zero-heat-flux (℃)	36.9 (36.6–37.1)	36.8 (36.8–37.0)	36.6 (36.5–37.0)	0.43
Temperature by urinary catheter (℃)	–	36.9 (36.7–37.3)	36.5 (36.5–37.0)	0.29
Heart rate (bpm)	71 (65–77)	64 (57–71)^+^	63 (57–83)	0.045
Systolic blood pressure (mmHg)	142 (134–164)	96 (92–111)^+^	107 (95–132)	0.006
Mean blood pressure (mmHg)	92 (90–101)	64 (62–74)^+^	73 (62–87)	0.006
Diastolic blood pressure (mmHg)	67 (60–73)	53 (47–56)^+^	54 (46–72)	0.015
SaO_2_ (%)	97.1(96.8–97.8)	99.4 (99.3–99.6)^+^	99.4 (99.3–99.6)^#^	< 0.0001
Cardiac output (l/min)	4.7 (4.3–4.9)	3.6 (3.1–4.5)	3.6 (2.9–4.5)	0.10
Cardiac index (l/min/m^2^)	2.8 (2.6–3.1)	2.1 (1.8–2.6)	1.9 (1.8–2.8)	0.10
Systemic vascular resistance index (dyne × sec × cm_5_ /m_2_)	2607 (2292–3171)	2438 (2114–2918)	2841 (2090–3007)	0.63
Stroke volume index (ml/beat/m^2^)	39.4 (34.6–40.6)	33.3 (30.7–40.6)	33.3 (28.1–37.9)	0.63
Bispectral Index value	–	52(49–56)	51(49–56)	–
Hemoglobin (g/dl)	13.3 (11.4–14.0)	11.7 (10.1–12.5)^+^	10.8 (9.9–12.3)^#^	< 0.0001
Lactate (mg/dl)	1.08 (0.84–1.44)	0.98 (0.87–1.57)	1.23 (1.06–1.35)	0.84
Oxygen content in arterial blood (ml/dl)	17.3 (14.6–18.8)	16.3 (14.2–17.2)^+^	14.8 (13.9–16.5)^#^	0.0002

Data are represented as the median (interquartile range) or number of patients. SaO_2_; Arterial oxyhemoglobin saturation, Oxygen content in arterial blood; Hemoglobin × SaO_2_ × 1.34 (ml/dl), ^+^*p* < 0.05 vs. T1, ^#^*p* < 0.05 vs. T1 (adjustment for multiple comparisons, Bonferroni)

In a cohort of 10 patients, we observed a statistically significant decrease over three time points in three key metrics: VO_2_/BSA, VCO_2_/BSA, and DO_2_/BSA. Specifically, the median VO_2_/BSA dropped from 130 (122–137) ml/min/m^2^ at T1 to 107 (81–133) ml/min/m^2^ at T2, and then further declined to 97 (93–121) ml/min/m^2^ at T3. This decline was statistically significant (*p* = 0.011). Similarly, the median VCO_2_/BSA decreased from 115 (105–129) ml/min/m^2^ at T1 to 90 (71–107) ml/min/m^2^ at T2, and decreased further to 81 (69–101) ml/min/m^2^ at T3. This trend was also statistically significant (*p* = 0.0020).

**Fig. 2:**
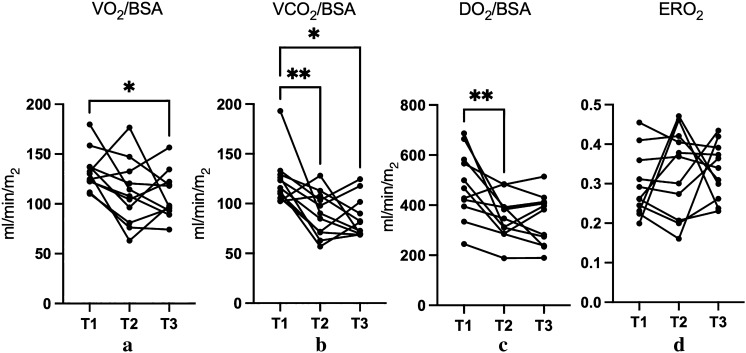
Results of VO_2_, VCO_2_, DO_2_, and ERO_2_. Before and after induction of general anesthesia, VO_2_, VCO_2_, and DO_2_ dropped, but there was considerable variation among the patients. ERO_2_ did not change appreciably. *p < 0.05, T1 vs. T3. **p < 0.05, T1 vs. T2. VO_2_, oxygen consumption; VCO_2_, carbon dioxide production; DO_2_, oxygen delivery; ERO_2_, oxygen extraction rate; T1, first time point; T2, second time point; T3, third time point; *BSA* body surface area

DO_2_/BSA followed a slightly different pattern. It decreased from a median of 467 (395–582) ml/min/m^2^ at T1 to 347 (286–392) ml/min/m^2^ at T2, but then increased slightly to 382 (238–414) ml/min/m^2^ at T3. This overall change was statistically significant (*p* = 0.0011). These results are visually represented in Fig. 2 and numerically detailed in Table 3.

**Table 3:** Values for the supply and demand of oxygen

	T1	T2	T3	*p*
Oxygen consumption/BSA (ml/min/m^2^)	130 (122–137)	107 (81–133)	97 (93–121)^#^	0.011
Carbon dioxide production/BSA (ml/min/m^2^)	115 (105–129)	90 (71–107)^+^	81 (69–101)^#^	0.0020
Oxygen delivery/BSA (ml/min/m^2^)	467 (395–582)	347 (286–392)^+^	382 (238–414)	0.011
Oxygen extraction ratio (%)	26.1 (23.0–35.9)	36.8 (20.6–42.0)	33.9 (26.1–39.1)	0.97
Double product (bpm × mmHg)	10,275 (8500–11620)	6141 (5063–7332)^+^	7150 (5082–9184)	< 0.0001
LaFarge–Miettinen oxygen consumption/BSA (ml/min/m_2_)	140 (136–146)	135 (134–142)^+^	140 (137–143)	0.045

Data are represented as the median (interquartile range)

*BSA* body surface area

^+^p < 0.05 vs. T1, ^#^p < 0.05 vs. T1 (adjustment for multiple comparisons, Bonferroni)

The analysis revealed a characteristic trend in the change in oxygen metabolism between the two groups divided by the VCO_2_ at T2. There were no significant differences in the patients’ backgrounds, such as age and sex, between these two groups (Online Resource 1). There were also no significant differences at T1 for hemodynamics (e.g., heart rate, blood pressure, cardiac coefficient), respiratory status (minute ventilation rate, respiratory rate), oxygen supply–demand balance indices (VO_2_, VCO_2_, DO_2_), and acid–base equilibrium (pH, bicarbonate) (Online Resources 2 and 3). In contrast, no significant differences were found at T2 for the circulatory dynamics; however, differences were found for respiratory status (tidal volume and minute ventilation volume) and acid–base equilibrium (bicarbonate). Regarding the oxygen supply–demand balance index, VO_2_ showed similar highs and lows as those for VCO_2_, but DO_2_ showed no difference between the two groups.

## Discussion

The key findings in this study were as follows:

1. Remimazolam for induction of general anesthesia led to significant reductions in VO_2_ and VCO_2_, and an initial drop in DO_2_, indicating suppressed metabolic activity and oxygen demand.
2. Patients stratified by VCO_2_ levels exhibited divergent patterns in respiratory parameters and acid–base status post-induction, despite comparable baseline characteristics.
3. While changes in VO_2_ paralleled those for VCO_2_, DO_2_ remained similar between the groups, implying remimazolam’s metabolic effects may depend on the patient’s underlying metabolic state.

These findings provide important insights into the metabolic alterations and compensatory oxygen supply–demand dynamics during induction of general anesthesia with remimazolam. These findings highlight the need for individualized anesthetic management strategies that account for the patient’s specific metabolic profile and oxygen demands.

Most of the previously published reports we collected examined oxygen supply–demand balance using inhaled anesthetics. Jakobsson et al. [32] reported a 33 ml/min/m^2^ (95% confidence interval, 28–38 ml/min/m^2^) reduction in VO_2_ in a systematic review. Crozier et al. [33] reported a 102 ml/min (32.1%) reduction in VO_2_ after induction of general anesthesia with midazolam. In this study, as in a previous study [18], both oxygen supply and VO_2_ decreased. However, in contrast to the previous study, in which both oxygen supply and VO_2_ decreased simultaneously, the decrease in VO_2_ was smaller than previously reported in this study, with decreases in VO_2_, VCO_2_, and DO_2_ of 23 ml/min/m^2^ (17.7%), 25 ml/min/m^2^ (21.8%), and 120 ml/min/m^2^ (25.7%), respectively. Consequently, the ERO_2_ ranged from 26.1% during wakefulness to 36.8% during general anesthesia.

DO_2_ dependence on VO_2_ occurs when DO_2_(crit) is below the established range [9, 22, 34]. During anesthesia and cardiac resuscitation, DO_2_(crit) in healthy awake patients ranges from 270 to 330 ml/min/m^2^ [7, 35, 36]. The mechanism by which ERO_2_ is modified and corrected operates when DO_2_ is maintained above the critical value [7–9]. DO_2_(crit) thresholds are also elevated by mitochondrial dysfunction in patients with sepsis and hyperlactatemia [37, 38]. In this study, we observed some cases in which DO_2_/BSA fell below 330 ml/min/m^2^ at T2, but we encountered no cases with an increased R/Q. However, we observed cases with an R/Q of > 1 at T1. This suggests that the effect of sympathetic dominance during arousal on the tissue oxygen supply–demand balance is greater than the effect of parasympathetic dominance due to general anesthesia [39].

The most important difference between the present study and previous studies is in the use of general anesthesia. Because of concerns that inhalation anesthetics would compromise the accuracy of the indirect calorimetry data, only intravenous anesthetics were used in the present study. To exclude the impact of changes in hemodynamics and oxygen supply caused by vasoconstrictor treatment, we also excluded patients who received vasoconstrictor treatment. Inhaled anesthetics have ischemia preconditioning effects [40], and the changes in VO_2_ and VCO_2_ likely differ between inhaled and intravenous anesthetics, including remimazolam, in terms of the DO_2_ decrease.

At the cellular and tissue levels, biological reactions to variations in DO_2_ are distinct. Hypoxic cells respond with a self-imposed program to cut energy consumption by directly sensing a fall in oxygen levels long before the adenosine triphosphate (ATP) pool is depleted and by shutting down non-essential cellular processes [3]. Therefore, VO_2_ may have declined in a DO_2_-dependent manner. Because R/Q did not differ between the two groups, the low VO_2_ and VCO_2_ levels in the low VCO_2_ group were within the range of physiological alterations and above the DO_2_(crit) level.

When comparing differences in the variables between the two groups, no differences were found in the awake group (Online Resource 1). Under general anesthesia, differences were observed in VO_2_ as well as high and low VCO_2_ values, but there were no significant differences in the hemodynamic parameters, such as blood pressure, heart rate, cardiac output, stroke volume, peripheral vascular resistance, or DO_2_. The partial pressure of end-tidal CO_2_ values did not differ between the two groups because minute ventilation changed in parallel with changes in VCO_2_; however, differences were observed in acid–base equilibrium. Therefore, the effect on acid–base equilibrium was thought to have been caused by the change in cellular CO_2_ production.

The reason for dividing the patients into two groups (high and low VCO_2_) stems from a clinical question. The clinical question is whether changes in oxygen supply may cause fluctuations in VCO_2_ during induction of general anesthesia (as inferred from the minute ventilation rate and end-tidal CO_2_), which will require adjustments in respiratory management. Because VCO_2_ should correlate with VO_2_ in all but extreme oxygen metabolic situations, we thought that the relationship between oxygen supply and VO_2_ was relevant. We considered this an important topic because the maintenance of homeostasis, including oxygen metabolism, is the anesthesiologist’s responsibility during general anesthesia. Although the patients in this study were scheduled for surgery and were in a stable general condition, the discovery that only some patients tended to have decreased VCO_2_ values was considered a new finding.

The body composition variables influencing VO_2_, such as circulating blood volume, body temperature and its distribution, and lean body mass, may have varied among the patients. Changes in body temperature are related to alterations in tissue heat content (i.e., VO_2_ changes) [41]. The induction of general anesthesia may have decreased the deep body temperature and raised the peripheral heat content in the extremities [42], thereby altering the oxygen supply–demand balance.

In one patient in this study, DO_2_ and VO_2_ increased during the induction of general anesthesia. The data analysis revealed a drop in blood pressure and pulse rate from T1 to T2. However, cardiac output rose while peripheral vascular resistance fell. The ZHF temperature at T1 was as low as 35.1 °C, whereas the ZHF temperature was 36.5 °C, and the bladder temperature was 34.2 °C, at T2. The ZHF temperature did not vary by more than 1.4 °C in any other patients. The rapid redistribution of temperature brought on by general anesthesia may have contributed to the increases in DO_2_ and VO_2_ at T2.

The limitations of our study are that the characteristics of the patient groups and research methods affected the results. First, regarding the uncertainty of the measurement method, only a single measurement was taken each time. Given that each measurement took approximately 20 min to complete, the time required for many measurements would have been clinically inappropriate. Second, the third measurement was taken after the commencement of surgery. The results might have been influenced by factors, such as epidural anesthesia, bleeding, and surgical invasion. However, no patients developed massive bleeding that required transfusion and vasoconstrictor treatment. Despite our utmost efforts to eliminate as many factors as possible that could impact the balance of oxygen supply and demand, this remains an important limitation of our study and may have affected the consistency of the measurement results. Third, the indirect calorimetry measurements during the waking state may have contained errors due to the omission of respiration-related leakage from the device gap.

The findings of this study have important implications for intensivists and anesthesiologists who administer general anesthesia. It is often challenging to determine the appropriate amount of oxygen required for each patient in clinical practice, and the respiratory and cardiovascular systems are typically managed based on the established safe zone. Importantly, further developments in the methodology of this study may facilitate predictions of future changes in VO_2_ after the induction of general anesthesia.

In conclusion, general anesthetic induction with remimazolam decreased VO_2_, VCO_2_, and DO_2_. The data suggest that some cases behaved differently in response to changes in the oxygen supply–demand balance despite having the same settings for breathing conditions.

## Data Availability

The data used and/or generated in this study are available upon reasonable request. Data sharing will be done after ensuring there are no issues with protecting personal information or intellectual property rights. If you wish to access the data, please contact the corresponding author.

## References

[1] Hirota K. Hypoxia-inducible factor 1, a master transcription factor of cellular hypoxic gene expression. J Anesth. 2002;16(2):150–9. 14517667 10.1007/s005400200011

[2] Semenza GL. Regulation of mammalian O2 homeostasis by hypoxia-inducible factor 1 annual review of cell and developmental biology. Annu Rev Cell Dev Biol. 1999;15:551–78. 10.1146/annurev.cellbio.15.1.551. 10611972 10.1146/annurev.cellbio.15.1.551

[3] Hochachka PW, Buck LT, Doll CJ, Land SC. Unifying theory of hypoxia tolerance: molecular/metabolic defense and rescue mechanisms for surviving oxygen lack. Proc Natl Acad Sci USA. 1996;93(18):9493–8. 8790358 10.1073/pnas.93.18.9493 PMC38456

[4] Soong JTY, Soni N. Circulatory shock. Med UKi. 2013;41(2):64–9.

[5] Cecconi M, De Backer D, Antonelli M, Beale R, Bakker J, Hofer C, Roman J, Alexandre M, Michael Pinksey R, Jean Louis T, Jean Louis V, Andrew R. Consensus on circulatory shock and hemodynamic monitoring. Task force of the European society of intensive care medicine. Intensive Care Med. 2014;40(12):1795–815. 25392034 10.1007/s00134-014-3525-z PMC4239778

[6] Dyson A, Ekbal N, Stotz M, Barnes S, Carré J, Tully S, Henderson S, Barret L, Singer M. Component reductions in oxygen delivery generate variable haemodynamic and stress hormone responses. Br J Anaesth. 2014;113(4):708–16. 24852502 10.1093/bja/aeu089

[7] Shibutani K, Komatsu T, Kubal K, Sanchala V, Kumar V, Bizzarri DV. Critical level of oxygen delivery in anesthetized man. Crit Care Med. 1983;11(8):640–3. 6409505 10.1097/00003246-198308000-00010

[8] De Backer D. VO2/DO2 relationship: how to get rid of methodological pitfalls? Intensive Care Med. 2000;26(12):1719–22. 11271076 10.1007/s001340000713

[9] Vincent JL. DO 2/VO 2 relationships. Funct Hemodynamic Monit Updat Intensive Care Emerg Med. 2005;42:1–2.

[10] Vincent JL, De Backer D. Oxygen transport - the oxygen delivery controversy. Intensive Care Med. 2004;30(11):1990–6. 15258731 10.1007/s00134-004-2384-4

[11] De Backer D. Lactic acidosis. Intensive Care Med. 2003;29(5):699–702. 12682722 10.1007/s00134-003-1746-7

[12] Dubin A, Pozo MO, Hurtado J. Central venous minus arterial carbon dioxide pressure to arterial minus central venous oxygen content ratio as an indicator of tissue oxygenation: a narrative review. Rev Bras Ter Intensiva. 2020;32(1):115–22. 32401981 10.5935/0103-507X.20200017 PMC7206946

[13] Dubin A, Ferrara G, Kanoore Edul VS, Martins E, Canales HS, Canullán C, Murias G, Pozo MO, Estenssoro E. Venoarterial PCO_2_-to-arteriovenous oxygen content difference ratio is a poor surrogate for anaerobic metabolism in hemodilution: an experimental study. Ann Intensive Care. 2017. 10.1186/s13613-017-0288-z. 28608134 10.1186/s13613-017-0288-z PMC5468362

[14] Kang AR, Lee J, Jung W, Lee M, Park SY, Woo J, Kim SH. Development of a prediction model for hypotension after induction of anesthesia using machine learning. PLoS ONE. 2020;15(4):1–17. 10.1371/journal.pone.0231172. 10.1371/journal.pone.0231172 PMC7162491 32298292

[15] Terao Y, Miura K, Saito M, Sekino M, Fukusaki M, Sumikawa K. Quantitative analysis of the relationship between sedation and resting energy expenditure in postoperative patients. Crit Care Med. 2003;31(3):830–3. 12626992 10.1097/01.CCM.0000054868.93459.E1

[16] Vernon DD. effect of neuromuscular blockade on oxygen consumption and energy expenditure in sedated, mechanically ventilated children. Pediatr Crit Care. 2000;28(5):10–27. 10.1097/00003246-200005000-00051 10834713

[17] Bardutzky J, Georgiadis D, Kollmar R, Schwab S. Energy expenditure in ischemic stroke patients treated with moderate hypothermia. Intensive Care Med. 2004;30(1):151–4. 12955178 10.1007/s00134-003-1988-4

[18] Jakobsson J, Vadman S, Hagel E, Kalman S, Bartha E. The effects of general anaesthesia on oxygen consumption: a meta-analysis guiding future studies on perioperative oxygen transport. Acta Anaesthesiol Scand. 2018;63(2):144–53. 30238445 10.1111/aas.13265

[19] Schüttler J, Eisenried A, Lerch M, Fechner J, Jeleazcov C, Ihmsen H. Pharmacokinetics and pharmacodynamics of remimazolam (CNS 7056) after continuous infusion in healthy male volunteers part I. Pharma Clin Pharma Anesthesiol. 2020;4:636–51. 10.1097/ALN.0000000000003103 31972655

[20] Doi M, Morita K, Takeda J, Sakamoto A, Yamakage M, Suzuki T. Efficacy and safety of remimazolam versus propofol for general anesthesia: a multicenter, single-blind, randomized, parallel-group, phase IIb/III trial. J Anesth. 2020;34(4):543–53. 10.1007/s00540-020-02788-6. 32417976 10.1007/s00540-020-02788-6

[21] Rex DK, Bhandari R, Desta T, DeMicco MP, Schaeffer C, Etzkorn K, Barish CF, Pruitt R, Cash BD, Quirk D, Tiongco F, Sullivan S, Bernstein D. A phase III study evaluating the efficacy and safety of remimazolam (CNS 7056) compared with placebo and midazolam in patients undergoing colonoscopy. Gastrointest Endosc. 2018;88(3):427-437.e6. 10.1016/j.gie.2018.04.2351. 29723512 10.1016/j.gie.2018.04.2351

[22] Suga H, Hisano R, Goto Y, Yamada O, Igarashi Y. Effect of positive inotropic agents on the relation between oxygen consumption and systolic pressure volume area in canine left ventricle. Circ Res. 1983;53(3):306–18. 6883652 10.1161/01.res.53.3.306

[23] Jakobsson J, Norén C, Hagel E, Kalman S, Bartha E. Peri-operative oxygen consumption revisited: an observational study in elderly patients undergoing major abdominal surgery. Eur J Anaesthesiol. 2021;38(1):4–12. 32858583 10.1097/EJA.0000000000001302

[24] Crystal GJ, Silver JM, Salem MR. Mechanisms of increased right and left ventricular oxygen uptake during inotropic stimulation. Life Sci. 2013;93(2–3):59–63. 10.1016/j.lfs.2013.05.011. 23747965 10.1016/j.lfs.2013.05.011

[25] Hahm TS, Jeong H, Ahn HJ. Systemic oxygen delivery during one-lung ventilation: comparison between propofol and sevoflurane anaesthesia in a randomised controlled trial. J Clin Med. 2019;8(9):1438. 31514342 10.3390/jcm8091438 PMC6780591

[26] Acute Respiratory Distress Syndrome Network Roy G Brower Michael A Matthay Alan Morris David Schoenfeld B Taylor Thompson Arthur WheelerAcute Respiratory Distress Syndrome Network Roy G Brower Michael A Matthay Alan Morris David Schoenfeld B Ta AW. Ventilation with lower tidal volumes as compared with traditional tidal volumes for acute lung injury and the acute respiratory distress syndrome. N Engl J Med. 2000. 10.1056/NEJM200005043421801. 10.1056/NEJM200005043421801 10793162

[27] Frayn KN. Calculation of substrate oxidation rates in vivo from gaseous exchange. J Appl Physiol. 2016;121(6):628–34. 10.1152/jappl.1983.55.2.628 6618956

[28] Jorgensen CR, Wang K, Wang Y, Gobel FL, Nelson RR, Taylor H, Frank Gams R, JohnVilandre E. Effect of propranolol on myocardial oxygen consumption and its hemodynamic correlates during upright exercise. Circulation. 1973;48(6):1173–82. 4762475 10.1161/01.cir.48.6.1173

[29] LaFarge CG, Miettinen OS. The estimation of oxygen consumption. Cardiovasc Res. 1970;4(1):23–30. 10.1093/cvr/4.1.23. 5416840 10.1093/cvr/4.1.23

[30] Badal JJ, Loeb RG, Trujillo DK. A simple method to determine mixed exhaled CO_2_ using a standard circle breathing circuit. Anesth Analg. 2007;105(4):1048–52. 17898386 10.1213/01.ane.0000280484.81216.76

[31] Kligfield P. Heart disease: a textbook of Cardiovascular Medicine, 5/E, edited by Eugene Braunwald, W.B. Saunders, Philadelphia (1997) 2143 pages, illustrated, $125.00 ISBN: 9‐7216‐5666‐8. Clin Cardiol. 1998;21(2):147–8. 10.1002/clc.4960210223.

[32] Jakobsson J, Vadman S, Hagel E, Kalman S, Bartha E. The effects of general anaesthesia on oxygen consumption: a meta-analysis guiding future studies on perioperative oxygen transport. Acta Anaesthesiol Scand. 2019;63(2):144–53. 30238445 10.1111/aas.13265

[33] Crozier TA, Langenbeck M, Müller J, Kietzmann D, Sydow M, Kettler D. Total intravenous anaesthesia with sufentanil-midazolam for major abdominal surgery. Eur J Anaesthesiol. 1994;11(6):449–59. 7851351

[34] Weg JG. Oxygen transport in adult respiratory distress syndrome and other acute circulatory problems: relationship of oxygen delivery and oxygen consumption. Crit Care Med. 1991;19(5):650–7. 2026027 10.1097/00003246-199105000-00011

[35] Lieberman JA, Weiskopf RB, Kelley SD, Feiner J, Noorani M, Leung J, Pearl T, Maurene V. Critical oxygen delivery in conscious humans is less than 7.3 ml O_2_ kg^−1^ min^−1^. Anesthesiology. 2000;92(2):407–13. 10691227 10.1097/00000542-200002000-00022

[36] Smoor RM, van Dongen EPA, Verwijmeren L, Schreurs IAAM, Vernooij LM, van Klei WA, Noordzij PG. Critical oxygen delivery threshold during cardiopulmonary bypass in older cardiac surgery patients with increased frailty risk. Eur J Cardio Thorac Surg. 2021;00:1–8. 10.1093/ejcts/ezab396 34448850

[37] Protti A, Fortunato F, Monti M, Vecchio S, Gatti S, Comi GP, Giuseppe RD, Gattinoni L. Metformin overdose, but not lactic acidosis per se, inhibits oxygen consumption in pigs. Crit Care. 2012;16(3):R75. 22568883 10.1186/cc11332 PMC3580617

[38] Brody JS. Oxygen uptake supply dependency. Effects of short-term dobutamine infusion. Am Rev Respir Dis. 1990;4(3):1–5. 10.1164/ajrccm/142.6_Pt_2.S2 2368971

[39] Yilmaz B, Göktepe S, Yaşar E, Kesikburun S, Adigüzel E. The effect of autonomic nervous system dysfunction on oxygen consumption during daily living activities in patients with spinal cord injury. Spinal Cord. 2017;55(3):300–3. 27431660 10.1038/sc.2016.111

[40] Chen S, Lotz C, Roewer N, Broscheit JA. Comparison of volatile anesthetic-induced preconditioning in cardiac and cerebral system: molecular mechanisms and clinical aspects. Eur J Med Res. 2018;23(1):1–10. 10.1186/s40001-018-0308-y. 29458412 10.1186/s40001-018-0308-y PMC5819224

[41] Baraka A. Influence of surface cooling and rewarming on whole-body oxygen supply-demand balance. Br J Anaesth. 1994;73(3):418–20. 7946874 10.1093/bja/73.3.418

[42] Matsukawa T, Sessler DI, Sessler AM, Schroeder M, Ozaki M, Kurz A, Christi C. Heat flow and distribution during induction of general anesthesia. Anesthesiology. 1995;82(3):662–73. 7879935 10.1097/00000542-199503000-00008

